# No Advantage for Separating Overt and Covert Attention in Visual Search

**DOI:** 10.3390/vision4020028

**Published:** 2020-05-18

**Authors:** W. Joseph MacInnes, Ómar I. Jóhannesson, Andrey Chetverikov, Árni Kristjánsson

**Affiliations:** 1 School of Psychology, National Research University Higher School of Economics, Moscow 101000, Russia; ak@hi.is; 2 Vision Modelling Lab, Faculty of Social Sciences, National Research University Higher School of Economics, Moscow 101000, Russia; 3 Icelandic Vision Laboratory, Department of Psychology, University of Iceland, 102 Reykjavik, Iceland; oij1@hi.is; 4 Donders Institute for Brain, Cognition, and Behaviour, Radboud University, 6525 EN Nijmegen, The Netherlands; a.chetverikov@donders.ru.nl

**Keywords:** search, gaze-contingent, attention, covert, eye-movements

## Abstract

We move our eyes roughly three times every second while searching complex scenes, but covert attention helps to guide where we allocate those overt fixations. Covert attention may be allocated reflexively or voluntarily, and speeds the rate of information processing at the attended location. Reducing access to covert attention hinders performance, but it is not known to what degree the locus of covert attention is tied to the current gaze position. We compared visual search performance in a traditional gaze-contingent display, with a second task where a similarly sized contingent window is controlled with a mouse, allowing a covert aperture to be controlled independently by overt gaze. Larger apertures improved performance for both the mouse- and gaze-contingent trials, suggesting that covert attention was beneficial regardless of control type. We also found evidence that participants used the mouse-controlled aperture somewhat independently of gaze position, suggesting that participants attempted to untether their covert and overt attention when possible. This untethering manipulation, however, resulted in an overall cost to search performance, a result at odds with previous results in a change blindness paradigm. Untethering covert and overt attention may therefore have costs or benefits depending on the task demands in each case.

## 1. Introduction

The visual field contains, at any given moment, far more information than our visual system can process in detail. We therefore use attention to select items for further processing that are of potential importance [1,2] (see [3] for a review). This may have costs, however, as unattended items can often go unnoticed, and we can miss surprisingly salient events in our visual field if our visual attention is not directly applied to them [4,5,6]. At the same time, other stimuli can break through the clutter and capture our attention, even when this goes against our goals [7,8]. 

### 1.1. Overt and Covert Attention

The fovea centralis contains the highest density of cones on the retina, and is responsible for our high-resolution vision. The locus of attention often overlaps with our center of gaze, and it is natural to ensure that the location or item that we wish to scrutinize falls on the fovea. We move our eyes roughly three times a second to overtly shift our center of gaze to the attended location. However, we can also attend to locations in space without fixating them directly, and such covert orienting also produces behavioral changes at the attended location [9]. Such covert attending was probably first systematically investigated by Hermann von Helmholtz. He used a darkened room where only a small pinhole was visible on which he remained fixated [10]. Helmholtz discovered that he was able to select a region outside of the center of his gaze, and pay specific attention to that region. When the room was briefly illuminated with a flash, he was able to recognize letters in that region even though his gaze was centered elsewhere, while other letters at unattended locations were a blur. This revealed the existence of an attentional mechanism that was spatially selective but which did not involve eye movements and increased visual resolution at the attended location. 

Other evidence suggests that attention and eye movements are tightly coupled, so that attention can be typically expected to move to the locus of our gaze [11,12,13]. In fact, attention has been proposed to be intrinsically coupled with the oculomotor system (oculomotor readiness [14]; premotor theory [15]; see review in [16]). However, the relationship between attention and eye movements is probably not as all-or-none, as is often implied [17,18,19,20,21]. Although attention and eye movement networks do have significant overlap [22,23,24,25], the differences are often as telling as the similarities. The frontal eye fields (FEF) are involved in both attention and eye movements with single-cell stimulation, producing saccades with high-intensity stimulation and attention benefits with low-intensity stimulation [26]. Similarly, transcranial magnetic stimulation (TMS) to the FEF disrupts saccadic programming [27] and spatial attention in cueing [28] and searching [29]. Within the FEF, however, different populations mediate covert and overt attention [19], and may control attention and saccades independently [30]. Additionally, the findings of [31] suggest that endogenous saccade preparation is, on its own, not sufficient to observe well-known attention effects like inhibition of return (IOR). In summation, while attention and eye movements often work together, they reflect separate mechanisms and can therefore be uncoupled.

Models of vision in general and visual search in particular have used various approaches to the role of attention. Classic models assume that covert attention leads overt attention in the form of fixations on high-salience areas [32] (see [33] for a review), and these models are strongly rooted in theory (feature integration theory (FIT) [34]) for difficult searches. These theories have led to a focus on items, set size, and item features as the fundamental experiment manipulations. An alternate account of searching has been put forward by [35], where fixations are proposed as the primary descriptive unit of search performance. Since real-world searching rarely contains easily discriminable objects, it may be desirable to describe a framework where covert attention is deployed in parallel within each fixation. The authors suggest that scene difficulty may be included as a variable functional viewing field (FVF) centered around each fixation [36]. Fixation selection outside of this FVF may use an exploration mechanism that calculates expected information in new areas [37]. Regardless of the theory, however, visual exploration relies on an interplay between overt and covert attention.

### 1.2. Untethering Overt and Covert Attention

Paradigms that enable the untethering of covert and overt attention while observers can still move their eyes have not been available in the literature. Studies of covert attending in the literature typically require restricting gaze to fixation combined with a peripheral task, as in the example of Helmholtz described above (see, e.g., [9,38,39,40], but may also involve manipulating the amount of peripheral information available to the participant through gaze-contingent displays [41,42,43]. 

However, we have recently introduced a paradigm that allows a dissociation between covert and overt attention. We compared covert and overt contributions to a change blindness task, using mouse-contingent and gaze-contingent displays [44]. Observers had to judge whether a change occurred somewhere in an array of objects between two alternating presentations of the array. The array was covered with a grey mask, but observers could uncover portions of the array of stimuli through a Gaussian-shaped aperture by moving their gaze or by moving a mouse. While the gaze-contingent condition tethered the (covertly) viewable area to the center of gaze, the mouse-contingent condition allowed participants to untether the covert viewing area from their focus of gaze, while keeping the overall viewable area size the same. Participants were still able to move their eyes freely in the mouse-contingent condition, but it had no influence on the visible area. Using this approach, Chetverikov et al. investigated the potential costs and benefits of allowing observers to untether the two forms of attention. The results were that observers searched for the changing item differently when they could deploy covert attention separately from gaze position, in a seeming effort to use covert attention. Importantly, change detection was faster when overt and covert attention were uncoupled, indicating that there were benefits to this untethering of attention and gaze.

### 1.3. Current Aims

Attention has been studied extensively in various visual search tasks [45,46,47,48]. Here we attempt to address the question of what contribution covert attention makes to visual search, and when it can be allocated independently of overt attention. If voluntary attention can be applied both overtly and covertly, we can expect that performance on tasks like visual search might benefit under conditions where the two can be deployed separately, compared to when the two maintain a single focal point. 

**Figure 1:**
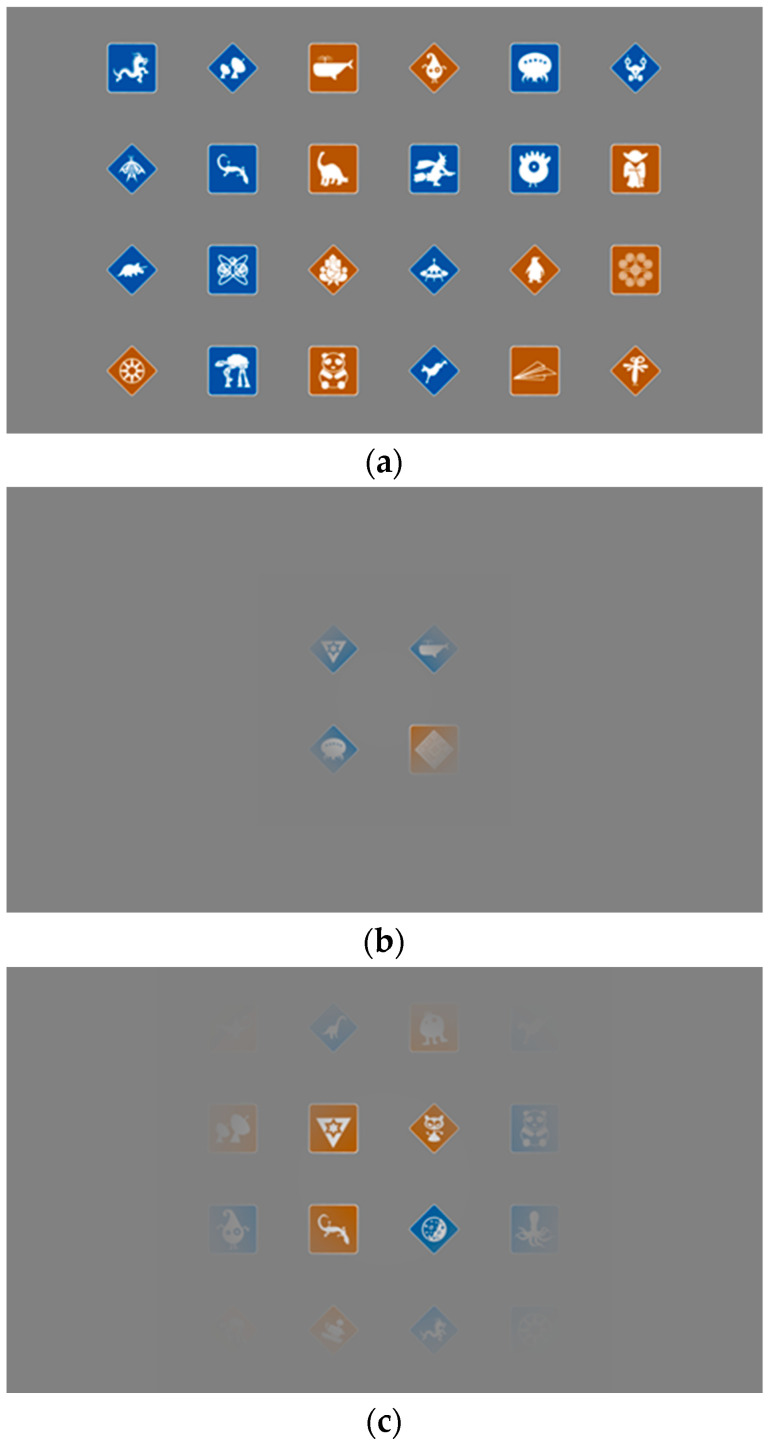
Sample stimuli used in the experiment. The two colors and two shapes were equally likely on every trial, but each item had a unique symbol within a trial. (**a**) Example visible area for fully visible, (**b**) example area for a 5 degree gradient aperture, and (**c**) example area for a 9 degree gradient aperture.

We used visual search displays where observers had to search for a target with a certain shape and color and a specific symbol within the shape. Examples of such “alien traffic sign” stimuli are shown in Figure 1. The symbol of the search target was unique within each trial, allowing for a straightforward search strategy; however, the color and shape of the search target were shared by some of the distractor stimuli. This allowed participants to use these features to guide the search and provided us with new measures of the degree to which observers used this guidance. Twenty-four such items appeared on the screen, but were covered by a grey mask that could be revealed through an aperture controlled by (1) gaze movement or (2) the movement of a mouse cursor. That is, the visible area for all trials was restricted and contingent on either mouse position or gaze position. The latter, mouse-contingent condition in particular allowed the uncovering of items independent of eye movements, while otherwise keeping the two conditions as similar as possible. We used two aperture sizes: either 5 or 9 degrees of visual angle.

In our gaze-contingent paradigm, moving the gaze around moved the mask. We compared such a paradigm with our newly designed mouse-contingent paradigm [44], where the visual display changes according to the real-time location of a user-controlled mouse cursor, allowing comparison of visual search performance with mainly overt attention (gaze-contingent display) and untethered overt and covert attention (mouse-contingent display). Our untethering manipulation does allow separate control of the gaze and visible area, but within some limits. First, it does not force participants to dissociate the overt and visible covert areas, but simply allows them to do so if they choose. Second, the limited visible area in the mouse-contingent condition restricts the useful screen to an area around the mouse aperture. This will likely mean that the center of covert and overt attention could be corelated to some extent, even when untethered. 

We ask the following questions: firstly, do observers try to utilize this covert attention, which is simulated with mouse movements, that observers control themselves during a visual search task, or do observers volitionally “tether” the gaze and mouse positions? Secondly, can observers use this possibility of untethered covert and overt attention to improve their performance during a visual search task? We assessed this by looking at performance (response time and accuracy) and tracking observers’ gazes as they performed the visual search task. Thirdly, what insights can our paradigm provide about the contribution of overt and covert attention to visual search performance? Our methodology is agnostic as to whether search is grounded in item properties or fixations, since our apertures will likely limit the FVF in either case. Separating the aperture from the center of the gaze will untether covert attention regardless of whether it extends to the full screen or is centered as an FVF on each successive fixation. We also include a combination of performance measures (accuracy, search time, and fixation analyses) to address both potential bases for visual search [35].

## 2. Methods

Twenty participants were recruited from the University of Iceland student population and received course credit for their participation. All procedures performed in studies involving human participants were in accordance with the ethical standards of the institutional and/or national research committee, and with the 1964 Helsinki declaration and its later amendments or comparable ethical standards. Informed consent was obtained from all individual participants included in the study. One participant was removed from analyses because of below-chance-level performance (17% correct) in the fully visible condition (likely due to reversing the response keys). The study was conducted in line with the requirements of the appropriate ethics committee, and all participants signed informed consent. 

The movements of the right eye were tracked at 500 Hz with an EyeLink 1000+. The stimuli were presented on a 24 inch-wide monitor (BenQ, model XL24112) with a resolution of 1920 × 1080 pixels, running at 60 Hz. The distance from the eye to the screen was 100 cm. The computer used to control the presentation had an Intel Core i7 4 GHz processor and 8 GB RAM, running Windows 7 Professional 64-bit, and MATLAB with Psychophysics toolbox 3.0 [49] was used for programming the experimental displays. Head movements were restricted with a head and chin rest.

Participants were shown a target image at the start of each trial, and their task was to search for that unique target in a four-row by six-column matrix of items, and respond with a keyboard press whether the target was present or absent. The search items were chosen from the set used by Chetverikov [44] (see Figure 1 for examples), and could vary by shape (diamond or square), color (blue or brown), and symbol (64 possible drawn targets). The target and distractors were randomly drawn from the full set of images (64), colors (two), and shapes (two) for each trial. Selection was without replacement for a given trial, but with replacement between trials for a given block, meaning that there was a small chance that a symbol could repeat as the target within a block. The target symbol was unique within a trial when present, meaning the target symbol would never appear as a distractor with the non-target color or shape. Each trial was initiated by the participant with a button press and drift correction while looking at a central fixation. This sample target was shown complete with shape, color, and symbol information, even though it was possible to find the target by symbol alone. Mask, aperture, and trial stimuli representing the full search array were then presented in a four-row by six-column grid (See Figure 1). The width and height of each stimulus was 2 degrees visual angle (dva), with 2 dva spacing between each row and column. Target-absent trials (66.7% of each block) had equal likelihoods of each color and shape, and the set of symbols was randomly drawn from the full set, with the stipulation that symbols were not repeated regardless of color and shape. Target-present trials (33.3%) were similar, except that a random item was replaced with the actual target. Although the original intent was to present 50% target-present trials, a coding error resulted in the biased percentages reported here. No other problems resulted from this error, and both colors and shapes were equally likely as distractors on every trial. The non-duplication of symbols within each trial meant that participants could employ a strategy of searching a subset of the items presented based on color and/or shape, but they could also choose to ignore this information and perform the search based on the symbol alone. A trial lasted for a maximum of 8 s, or until the participant responded with a key press (left/right arrow for target present/absent).

All participants contributed data to five different blocks of trials, with 72 trials per block. The viewable search area differed for each block, with one having the full search array visible throughout each trial and the other four having only part of the screen visible through a participant-controlled aperture. These four aperture blocks consisted of each combination of two factors: aperture size (5 or 9 degree diameter visible), and aperture control (gaze or mouse). The visible area of the aperture involved a Gaussian gradient (see Appendix A for specific parameters) toward the edges, so that an additional one degree was partially visible to give the aperture a softer edge (see Figure 1b). Gaze aperture control fixed the center of the aperture to the participants’ gaze location, while the mouse control allowed a separation of gaze and aperture by controlling its center with the mouse. The fully visible condition was run first for all participants, in order to familiarize them with the task and search stimuli, while four aperture blocks were presented afterwards, according to a Latin square design. Each aperture block was also preceded by 10 practice trials, to allow observers to adapt to the various aperture controls. Aperture location was determined by the experiment code just prior to each monitor frame refresh (60 Hz), and redrawn with its center at the most recent location of the participants’ gaze or mouse.

## 3. Results

Our primary performance measures of visual search performance were accuracy of response and response times for trials with correct responses. We used these measures to determine the benefit, if any, of untethered covert attention in the mouse-contingent condition. Additionally, we calculated a number of eye-tracking metrics, in order to determine whether there was any evidence that participants chose to or were able to deploy such a strategy of untethering covert and overt attention. 

Response accuracy was analyzed with a generalized linear model (binomial family) using the lme4 package [50]. Statistical significance of the effects was estimated through model comparison using ANOVA χ2 tests. The subject was included as a random factor, with target information (color, shape, and symbol) tested as both intercepts and slopes. We anticipated potential interactions between aperture type and aperture size, so both variables were tested as fixed effects in addition to whether the target was present on that trial. Only the intercept of probe shape (χ2 (1) = 21.7; *p* < 0.001) improved the fit of the model (color – *p* = 0.42; "symbol” in the model was overfit/singular). In reference to the aperture contingency variables, “fully visible” refers to the condition where the full screen is visible throughout the trial. 

**Figure 2:**
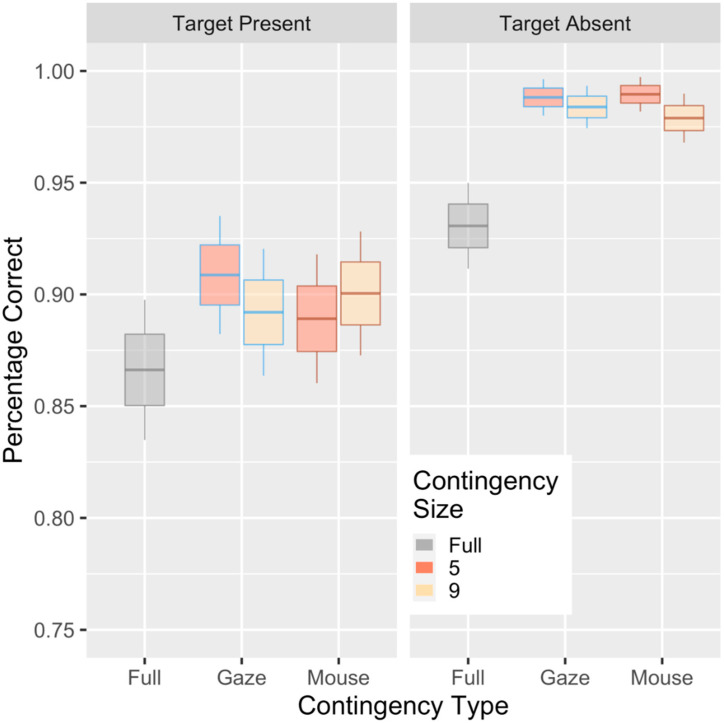
Accuracy for the base condition, as well as the mouse- and gaze-contingence conditions, shown as a function of whether a target was present or absent and the size of the aperture. Box plots represent category mean (center), standard error (box), and 95% CI (whisker).

The accuracy at the reference level (fully visible display and target present) was 86.1%. Target-absent trials were significantly more accurate (intercept + 8.6%; χ2 (1) = 175; *p* < 0.001) and they had different slopes (χ2 (2) = 55.9; *p* < 0.001). There was also a target present by contingency type interaction (χ2 (4) = 60.4; *p* < 0.001), with both contingent conditions gaining additional accuracy over the fully visible display when the target was absent (see Figure 2). Including other interactions, the main effects caused a failure of the model to converge, and tested at *p* > 0.20. The final model for accuracy was *(correct ~ Contingency:ProbeTrial + ProbeTrial + ProbeShape + (1+ ProbeTrial |Subject)*) in the lme4 syntax. A second model, removing the “fully visible” condition, showed only a main effect of whether a target was present or not with no significant interactions. The high accuracies in the reduced (aperture) viewing conditions suggest a speed/accuracy trade-off, but this was only in contrast to the fully visible condition, and not within the various different apertures. Analyses of d’ for the different contingencies also showed the high sensitivity (overall d’ of 3.3; standard error (SE) = 0.14). There was no difference between the contingent conditions (χ2 (2) = 3.4; *p* = 0.19), although the mouse condition was numerically the highest value (d’ = 3.5) Due to the high accuracies and lack of key interactions, we decided to focus primarily on response time as our primary measure of performance.

**Figure 3:**
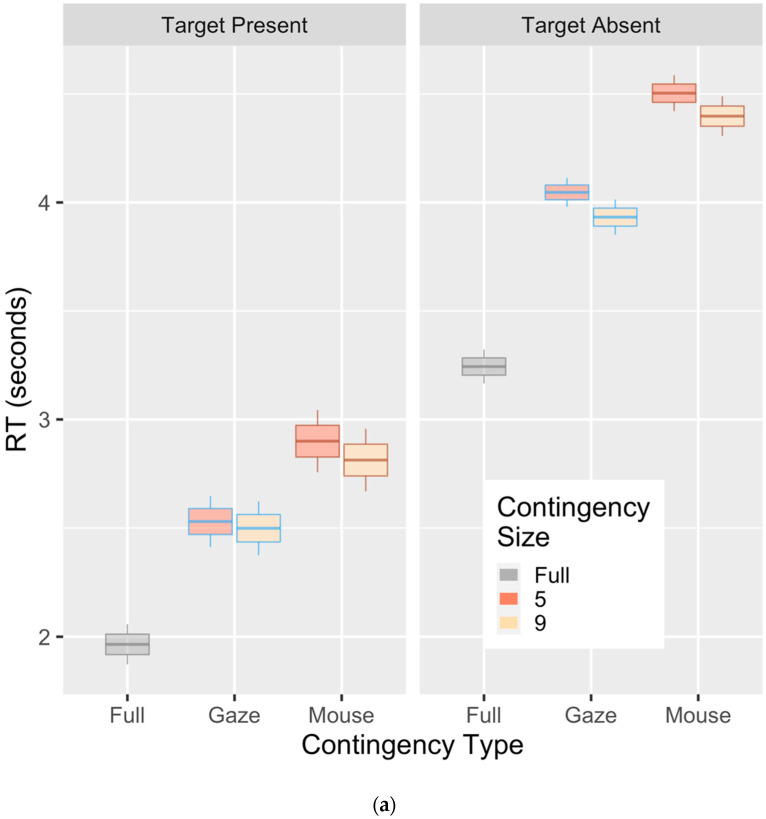

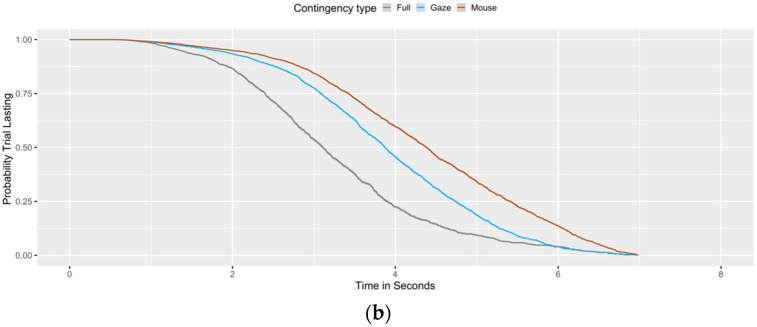
Overall search performance. (**a**) There were clear costs of introducing a contingent aperture as compared to fully visible conditions. As predicted, there was also a cost for a small (5 dva) compared to a large (9 dva) aperture. The additional cost of the mouse contingency over the gaze contingency was opposite to our predicted benefit of untethering mouse position and gaze. (**b**) The survival plot for target-present trials shows the probability of a trial continuing (due to the target not being found yet) past a certain time for each of the three conditions. Gaze-contingent trials diverge from mouse-contingent trials prior to the 2 second mark, and their advantage continues throughout. Box plots represent category mean (center), standard error (box), and 95% CI (whisker).

Response times (RTs) were analyzed with the same variable selection as accuracy, though the fully visible trials were removed and analyzed with the Gaussian lme4 family. The fully visible trials are plotted (Figure 3) but removed from analyses, due to their obvious performance advantage and since they do not have different aperture sizes for exploring our key interactions. Alternate models were compared using a chi-square test, in order to determine if additional variables significantly improve the overall model fit. The RT at model intercept (gaze-contingent condition, 5 dva) was 2.64 s (SE = 0.10) We observed the target presence intercept to be a main effect (χ2(1) = 54.7; *p* < 0.001), with responses on target-absent trials being 1.47 s slower than on target-present trials (*SE* = 0.10 s), as well as individual slopes for presence and absence (χ2(2) = 1593; *p* < 0.001). The key main effects were significant: contingency type (χ2(1) = 153; *p* < 0.001), with slower responses in the mouse-contingent than the gaze-contingent condition (+0.34 s, SE = 0.05); and aperture size, with faster responses (χ2 (1) = 7.6; *p* = 0.006) for the 9 dva aperture than the 5 dva one (−0.09 s; SE = 0.03). The interactions of probe trials and contingency was also significant (χ2 (1) = 11.5; *p* = 0.003). Additional interactions failed to improve the model fit (all *p* > 0.50), resulting in a final model (RT ~ ProbeTrial*Contingency+ ContingentSize+ +ProbeShape + (1+ ProbeTrial | Subject)) in the lme4 syntax. It should be noted here that the effect of the mouse-contingent condition was in the opposite direction than predicted, with a 340 ms cost of untethered covert attention. This also contrasts with our previously found benefits from the change blindness paradigm. This advantage for the tethered condition starts as early as one or two seconds, and continues throughout a trial (see Figure 3b). The RT analyses also reveal that there is a speed/accuracy trade-off in the fully visible condition, with relatively higher error rates and faster RTs in the target-present condition. We did not observe the same trade-off in the aperture conditions, since the accuracy scores were near ceiling level. 

### Evidence of Untethered Covert Attention

To determine if participants made any attempt to untether covert attention from the locus of gaze, we examined a number of mouse and gaze patterns. All analyses were run with linear mixed-effects regression, as described above, and included the same random effects. Gaze behavior was similar on target-present and -absent trials (see [51,52]), and will therefore be excluded for most models. We should note that just because the mouse contingent condition allows the aperture center to diverge from the center of the gaze, this does not guarantee that participants would adopt such a strategy. If participants adopted a strategy where they untethered the aperture to increase the use of covert attention, we would expect the gaze–mouse distance to increase. This would indicate that the mouse location is strategically separated from the centre of gaze, as opposed to observers matching the focus of gaze and aperture as best they could. We suggest at least 2 dva at minimum, which would place the center of the aperture outside the range of the fovea centralis. We also predicted that this distance would be greater for the 9 degree mouse aperture than with the 5 degree mouse aperture, since a larger potential area for covert attention was available. 

The average Euclidean distance between gaze and mouse was 3.15 dva (SE = 0.08) in the 5 dva aperture condition. Including the size of the aperture improved the fit of the model (χ2 (1) = 150, *p* < 0.001) and resulted in an increase of Euclidean distance of 0.28 dva (SE = 0.02) for the larger aperture. This result suggests that observers attempted to use this option of untethered covert attention.

**Figure 4:**
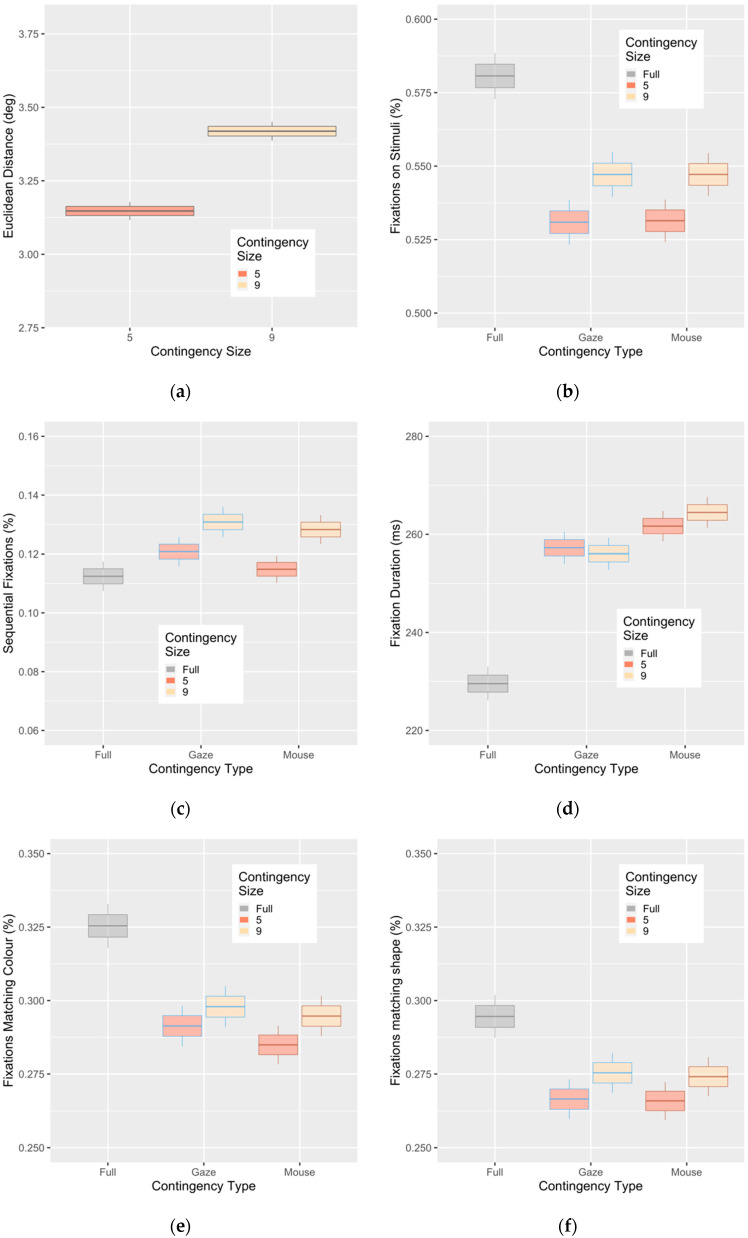
Metrics to determine if an effective covert strategy was used, including (**a**) the distance between mouse and gaze centers in the mouse-contingent condition; (**b**) the likelihood of fixations directly on stimuli (percentage from all fixations); (**c**) the likelihood of an adjacent stimulus being directly fixated on; (**d**) average fixation duration; (**e**) the likelihood of fixating a stimulus of the same color as the target; (**f**) the likelihood of fixating on the same shape as the target. Box plots represent category mean (center), standard error (box), and 95% CI (whisker).

If the mouse-contingent manipulation results in an increased use of covert attention, then fewer fixations directly on stimuli could reflect greater reliance on covert attention. Similarly, when observers do fixate on stimuli, they may be less likely to do so when using a simple sequential scanning strategy similar to “reading” rows or columns. When we examined fixations that landed directly on stimuli, there was a main effect of aperture size, but it was only due to the fully visible condition, where participants were less likely to fixate on stimuli compared to the two aperture conditions (Figure 4b). There were neither interactions nor differences between aperture conditions and sizes. For the likelihood of fixating on a directly adjacent stimulus (representing a more sequential scanning strategy), we found aperture size to be a main effect, with observers more likely to adopt this sequential strategy with the 9 dva apertures (χ2 (1) = 20.6; *p* < 0.001). The difference between gaze and mouse aperture type was not significant (χ2 (1) = 3.5; *p* = 0.062), but the trend showed fewer sequential fixations in the mouse condition. 

Average fixation duration for a trial may reflect cognitive load during a task, but it may also reflect an increase in the use of covert attention, reflected as the number of visual items being processed [53]. We observed aperture type as a main effect (χ2 (1) = 12.4, *p* < 0.001), with fixations in mouse-contingent trials being 4 ms slower (SE = 2.2) than in gaze contingent trials (Figure 4d). Inclusion of contingency size (*p* = 0.62) and the type by size interaction (*p* = 0.63) did not improve the fit of the model. If this cost in fixation duration were caused by increased task difficulty, we would expect to see increased fixation durations for the smaller and more difficult 5 degree apertures, but there was no effect of aperture size and no interaction. 

Our stimuli were selected so that they would provide redundant information that could potentially be used to make the search more efficient. The color and shape information of the target are non-essential information for the task; however, one or both could be used to limit the number of potential stimuli that need to be inspected on a given trial. For example, if the search target was a blue (color) and square (shape), with a tree inside (symbol), then participants could deploy covert attention to preselect only blue or square items for further selection, performing a so-called “subset search” based only on these features [54,55]. As such, we suggest that increased fixations on items that match the color or shape of the target represent a successful attempt to deploy covert attention. In particular, if participants used covert attention to dismiss distractors based on color or shape, they could thus focus overt attention directly on the remainder for a more efficient subset search. For these analyses, we calculate the percentage of fixations that match the target color or shape out of all possible fixations. Note that color and shape percentages are not mutually exclusive, since a fixation may land on a stimulus that matches the target in both color and shape. For the conditions with apertures, participants were more likely to fixate on distractors with the same shape (χ2 (1) = 5.9; *p* = 0.015) and the same color (χ2 (1) = 5.8; *p* = 0.016) (Figure 4e,f). There was no effect of aperture type on shape (χ2 (1) = 0.05; *p* = 0.83), while there was a non-significant trend for color (χ2 (1) = 3.1, *p* = 0.08). Furthermore, the (non-significant) direction for both color and shape was for fewer matching fixations in the mouse-contingent condition, suggesting that if observers attempted to use covert attention to preselect subsequent stimuli, they were less successful at doing so.

## 4. Discussion

We used a new paradigm that enables the untethering of covert and overt attention to investigate visual search performance. We asked two main questions: (1) do observers deploy covert attention separately when given this possibility during visual search, and (2) if covert attention is untethered, does this improve their performance? Five experimental conditions in a visual search task were tested using a within-subject design, where the area of an aperture and the method of aperture control were manipulated. As expected, limiting the area of covert attention with the apertures influenced performance, with performance in the full-visible condition exceeding all aperture conditions, and performance with large apertures (9 dva) better than with small ones (5 dva). These results suggest that our contingent manipulation was successful at limiting the scope of covert attention, and that limiting covert attention negatively affected search performance. We also observed evidence for changes in the search strategy with the mouse-contingent aperture compared to the gaze-contingent one. Participants tended to maintain the aperture focal points more than 3 degrees away from the focus of gaze, and this separation increased with the larger apertures. Fixations were also longer in the mouse-contingent condition. Taken together, the evidence suggests that participants attempted to use the mouse aperture untethered from the focus of gaze. Contrary to our hypothesis, however, separate control of the aperture by using the mouse resulted in worse search performance, as measured by average response times during the search and fewer saccades to distractors with matching features. Survival analyses suggested that this disadvantage occurred early in the first second (Figure 3) of the search, and was maintained throughout the search duration. 

Covert attention improves search performance for both feature and conjunction searches [56] by increasing the rate by which information is processed at the attended location [40]. Covert attentional selection during serial conjunction search is likely directed by the frontal eye fields [57], with possible additional contributions from other networks, including the supplementary eye fields [23] and parietal areas [58]. Although the premotor theory of attention suggests that covert attention is tied to the same network as for generating eye movements, activity within the FEF for the two has been shown to be independent [30]. If the two networks can operate independently, then there is potentially a benefit to be gained if the locus of covert attention can be separated from the locus of gaze. In fact, in a previous study, with the change-blindness paradigm, we found such improved performance when participants were able to control a visible aperture separately from the center of gaze [44]. 

Although some tasks may benefit from an untethered spotlight of covert attention, our current visual search results align more closely with the active vision hypothesis [59]. The authors there proposed that covert attention has benefits for tasks like search and reading, not through gaze-independent selection of scene items, but primarily through the preview of subsequent fixation locations. For example, attention precedes a saccade to a spatial location [12], and participants report the time of saccade arrival up to 80 ms prior to their actual arrival when saccading to a spinning clock [60]. Covert attention towards peripheral locations is also diminished during a saccade, suggesting a common, limited attentional resource [61]. Remapping of a neuron’s receptive field just prior to a saccade (in lateral intraparietal cortex (LIP) [62]; and in FEF [63]) has been suggested as a neural mechanism to explain these behavioral results, and has also been suggested as the primary mechanism to maintain visual stability across eye movements [64,65]. 

We found no evidence that untethered covert attention led to a visual search benefit—in fact, we observed a consistent cost, possibly due to the extra demands of the mouse control. This presents a puzzle in light of our previous finding on performance in a change-detection task, where untethered attention led to improved performance [44]. We can speculate that it is detrimental to split attention between two loci in this visual search task, and that the attentional demands of the change blindness and visual search tasks differ. Attentional capacity is limited, and attempts at untethering may lead to costs in this case, ones that did not surface in the change detection task. One difference between the tasks is, for example, that a visual search can often confirm a target within a single instant, whereas change blindness relies on attention and comparison at the location over time (minimum of one flicker interval). Another key task difference is that during search, observers can have a template of target features that can be used to guide attention [66], whereas in change blindness, the task involves no such template, since what is changing is unknown. In our current experiment, for example, participants were able to guide the search using the known color or shape of the target stimuli. It is possible that this guidance belongs predominantly to the visual domain, but offers little or no advantage for hand movements. While there were a few additional methodological differences between the studies, the most obvious was perhaps that during the change detection task, the screen flickered, with blank screens interleaved with the stimuli. This means that there were regular onsets throughout the task that could attract covert attention in a way similar to more controlled cuing studies. Another key difference was that the change blindness task involved a dynamic aperture that would shrink over time while not in motion. This may have had an impact on participant strategy, encouraging more frequent aperture movements to reset the aperture size. For example, it is possible that an increased frequency of saccades to maintain aperture size loads a common attentional resource needed for covert, peripheral updating via remapping [61].

Although our results suggest that observers tried to use the mouse-contingent aperture condition to search independently of the locus of gaze, this resulted in fewer saccades to distractors that matched the target on one of the two additional features (shape and color). The idea that attention might be allocated differently depending on the task is not new—for example, inhibition of return is observed in search tasks, but not scene memorization or inspection [67], and eye movement patterns differ based on instruction [68,69,70,71]. Given the evidence here that participants actively tried to use the untethered aperture, we suggest that the difference in results for search and change blindness was due to differences in the task. Another potential reason why results differ between visual search and change blindness might be that the two reflect different levels of perceptual processing, and place different demands upon visual attention. Overall, we can conclude that the mouse-contingent task was sensitive to changes in the aperture area, suggesting that it carries promise for assessing covert attention. For untethering covert and overt attention, the next step will involve figuring out what explains the discrepancy.

## Data Availability

The datasets generated during and analyzed during the current study are available in the Open Science Framework (OSF) repository, https://osf.io/f9eqs/.

## Appendix A

MATLAB code for aperture:

apertureSize = floor(visArea × pixelsperdegree);

transLayer = 2;

[x,y] = meshgrid(−apertureSize: apertureSize, −apertureSize:);

maskblob=ones(2 × apertureSize + 1, 2 × apertureSize +1, transLayer) × grey;

% Layer 2 (Transparency aka Alpha) is filled with gaussian transparency

% mask.

xsd = apertureSize/2.2;

ysd = apertureSize/2.2;

maskblob(:,:,transLayer)=round(255 – exp (−((x/xsd).^2)−((y/ysd).^2)) × 255);
